# AI-Driven Adaptive Communications for Energy-Efficient Underwater Acoustic Sensor Networks

**DOI:** 10.3390/s25123729

**Published:** 2025-06-14

**Authors:** A. Ur Rehman, Laura Galluccio, Giacomo Morabito

**Affiliations:** Dipartimento di Ingegneria Elettrica Elettronica e Informatica (DIEEI), University of Catania & CNIT, Viale A. Doria 6, 95125 Catania, Italy; aziz.rehman@phd.unict.it (A.U.R.); giacomo.morabito@unict.it (G.M.)

**Keywords:** underwater communications, energy efficiency, CNN, software-defined radio systems, environmental sustainability

## Abstract

Underwater acoustic sensor networks, crucial for marine monitoring, face significant challenges, including limited bandwidth, high delay, and severe energy constraints. Addressing these limitations requires an energy-efficient design to ensure network survivability, reliability, and reduced operational costs. This paper proposes an artificial intelligence-driven framework aimed at enhancing energy efficiency and sustainability in applications of marine wildlife monitoring in underwater sensor networks, according to the vision of implementing an underwater acoustic sensor network. The framework integrates intelligent computing directly into underwater sensor nodes, employing lightweight AI models to locally classify marine species. Transmitting only classification results, instead of raw data, significantly reduces data volume, thus conserving energy. Additionally, a software-defined radio methodology dynamically adapts transmission parameters such as modulation schemes, packet length, and transmission power to further minimize energy consumption and environmental disruption. GNU Radio simulations evaluate the framework effectiveness using metrics like energy consumption, bit error rate, throughput, and delay. Adaptive transmission strategies implicitly ensure reduced energy usage as compared to non-adaptive transmission solutions employing fixed communication parameters. The results illustrate the framework ability to effectively balance energy efficiency, performance, and ecological impact. This research contributes directly to ongoing development in sustainable and energy-efficient underwater wireless sensor network design and deployment.

## 1. Introduction

Underwater acoustic sensor networks (UASNs) are available to revolutionize our ability to monitor marine ecosystems, conduct oceanographic studies, explore underwater resources, and enhance maritime safety. However, deploying effective underwater sensor networks, especially when using widespread acoustic technologies (In this work, we focus on acoustic underwater communication networks due to their widespread adoption in commercial scenarios), poses challenges due to propagation in the complex underwater medium exhibiting limited bandwidth, high delay, and signal attenuation. These limitations, combined with significant energy constraints at the equipment level, the impracticality of battery replacement, and finite operational lifespans, necessitate the design of energy-efficient strategies such as adaptive communication protocols, routing, scheduling, and cross-layer optimization to ensure network longevity.

A typical exemplary application for these technologies is non-invasive marine life monitoring, which requires novel approaches to detect and track underwater creatures while minimizing environmental impact [1] and energy usage, while also maintaining network functionality. Intelligent software-defined radio (SDR) systems powered by artificial intelligence (AI) can adapt transmission parameters based on the type of marine species monitored and identified in the surrounding of the sensor nodes, optimizing communication parameters for sustainable underwater sensing. Traditional sensing network architectures, which rely on the continuous transmission of raw sensor and image data to centralized surface stations for processing, amplify these limitations, leading to high energy consumption and reduced network longevity. Therefore, fundamentally new approaches to underwater sensing and communication are required to overcome these challenges and enable sustainable and scalable underwater monitoring.

In response to these challenges, AI emerges as an effective approach, decentralizing intelligence from centralized infrastructures directly to underwater sensor nodes. Employing lightweight AI inference models allows sensor nodes to locally classify marine species; instead of transmitting large files, these nodes share only classification outcomes, drastically reducing data volume. Through adaptive communication parameter selection, the system conserves energy and bandwidth while empowering real-time decision-making in dynamic underwater ecosystems. For instance, when rare species are detected, low-power modes with robust modulation (e.g., binary phase shift keying (BPSK)) and smaller packet length are employed to minimize interference. Conversely, for common species, higher-throughput modulation schemes (e.g., quadrature phase shift keying (QPSK)) with larger packet length and higher transmission power are selected to maximize bandwidth efficiency. This fusion of on-node inference and software-defined networking methodologies minimizes resource usage, energy consumption, and environmental impact.

To develop this system, a model was designed and simulated using GNU Radio to perform classification and experiment with different adaptive transmission parameters. The system performance was evaluated using key performance metrics such as energy consumption, bit error rate (BER), throughput, and delay. Tests validated the system ability to adapt its transmission strategy based on data semantics, demonstrating how AI-powered SDR systems can balance network performance, operational efficiency, and sustainability.

The rest of this paper is organized as follows. In Section 2, some related work is discussed. In Section 3, the considered system is illustrated. In Section 4, the channel model being adopted for characterizing the underwater medium is illustrated, while in Section 5, the performance results are reported. Finally, in Section 6, the conclusions are drawn.

## 2. State of the Art

In this section, we will describe relevant papers where the use of artificial intelligence techniques for the purpose of improving communication efficacy in underwater communication networks is proposed.

The challenges posed by the realization of an Internet of Underwater Things (IoUT) are numerous and mostly related to constrained energy consumption, the need for high bandwidth, and the unreliability of the medium. These factors are exacerbated by the limited energy resources, the difficulty of battery replacement, and the significant attenuation and scattering of signals in marine environments. In [1], all the abovementioned criticalities have been discussed in the perspective of understanding the potential exploitation of AI technology to transform the conservation, monitoring, and management of marine environments through automation. However, no specific focus on the optimization of communication mechanisms is presented, since the work disregards aspects associated with the design of on-node modulation, power control, or any other adaptive physical-level mechanisms, while presenting a more visionary discussion on potentials.

Indeed, the exploitation of AI-based tools can represent a successful way to cope with complexities of marine environments. In [2], the authors proposed a self-learning semantic framework for underwater communication that emphasizes transmitting semantically relevant information only. This approach reduces data redundancy and enhances communication efficiency by prioritizing information freshness and contextual importance; however, it does not incorporate any physical or link layer communication adaptivity, which makes the semantic methodology only aimed at data redundancy reduction.

Other studies have similarly leveraged network knowledge before or during the communication process. For instance, in [3], the authors utilized prior knowledge of underwater conditions to adjust network parameters and employ image compression to facilitate image transmission within bandwidth-constrained underwater environments.

In [4], a reinforcement learning approach was employed to improve image quality by observing both the received image quality and considering communication parameters at the sink node. This implies an appropriate choice of modulation and coding. Another semantic-based strategy is presented in [5], where images are transmitted in the form of textual descriptions and subsequently reconstructed at the receiver using text-to-image AI models. The primary objective of this method is to reduce transmission delay and enable real-time image communication in critical underwater conditions. In [6], the authors introduced a semantic approach targeting underwater monitoring applications. By employing deep learning-based encoder–decoder architectures within a semantic communication framework, they mitigated underwater channel impairments upon transmitting to offshore stations. For underwater optical communications, the authors in [7] developed a semantic communication framework capable of dynamically adapting transmission data rates in response to changing channel conditions.

From the point of view of distributed AI solutions, in [8], a distributed deep learning approach is used on the cloud infrastructure to train a fish segmentation model, thereby enhancing accuracy and efficiency for large marine datasets. After training, they deployed a compressed model on an edge device to enable real-time, energy-efficient inference in underwater environments, minimizing both delay and power consumption. In another study, the authors in [9] integrated distributed intelligence within a hybrid fish swarm optimization algorithm for underwater wireless sensor networks, allowing sensor nodes to autonomously optimize 3D coverage through local decision-making and information sharing. For on-node inference, the authors in [10] presented a battery-free machine learning pipeline for microcontroller units (MCUs), optimized using proxyless neural architecture search (ProxylessNAS) and static quantization. Their system, evaluated on the DeepFish dataset for underwater fish recognition, achieved 97.78% accuracy with only 0.57 mJ energy consumption, significantly reducing memory, runtime, and power requirements. However, the absence of a communication mechanism raises open questions regarding how inference outcomes are transmitted or accessed in deployment scenarios. Similarly, in [11], authors introduced a battery-free underwater sensing architecture that performs on-board CNN-based inference using energy harvested from underwater acoustics, with results transmitted via binary backscatter. While this system proves the feasibility of end-to-end battery-free inference, achieving 63% accuracy in the classification of real-time marine mammals, the communication strategy remains static and lacks adaptability to dynamic conditions.

In several studies, researchers have explored adaptive communication strategies in underwater acoustic networks, with a particular focus on modulation adaptation and power control, aiming to enhance energy efficiency and system performance under varying channel conditions. For example, in [12], the authors investigated adaptive power control in shallow-water communication channels using experimental data from the SPACE’08 experiment. They modeled the channel gain as a log-normal process and proposed a feedback-based linear prediction mechanism to adjust transmit power dynamically to maintain a target SNR, achieving an average reduction of 8.7 dB as compared to fixed-margin schemes. However, this approach is effective in improving energy efficiency, but it requires a periodic, error-free feedback of channel state information, which may be impractical in autonomous underwater deployments. On the other hand, the authors in [13], proposed an adaptive modulation approach for long-range underwater acoustic communications where the feedback from the receiver is not available due to long propagation delay. Their approach selects the best modulation scheme based on propagation modeling and a machine learning classifier trained on environmental parameters. They demonstrated approximately 80% accuracy in selecting the optimal modulation even under environmental mismatches. However, their method focuses on modulation adaptation and does not explore joint optimization with packet size, or power control, which could further improve performance.

All the above works exploit environmental knowledge, adaptive learning techniques, and lightweight inference to enhance underwater sensing; however, they do not consider the integration of classification with SDR-based communication adaptation in the underwater environment. In contrast, our framework enables on-node inference while dynamically tuning key transmission parameters such as modulation scheme, packet length, and transmission power based on the classification output, offering a more responsive and energy-efficient approach suited to real-world underwater environments.

## 3. System Description

**Figure 1:**
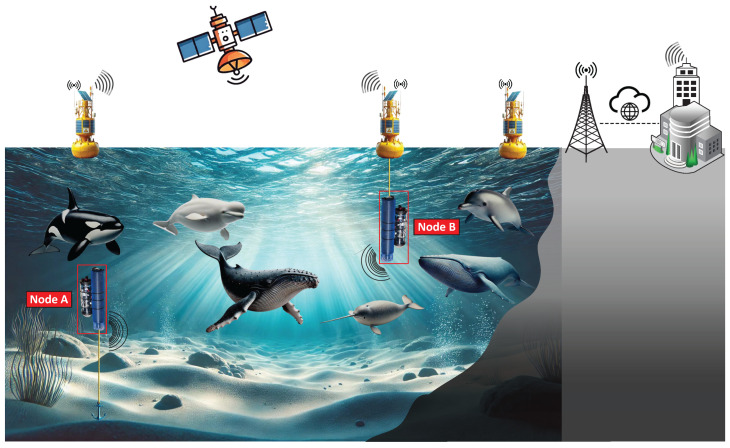
Scenario overview of underwater acoustic sensor network for monitoring.

We assume an underwater wireless sensor network where two nodes, A and B, are equipped with cameras, temperature sensors, light intensity sensors, and depth sensors. These nodes monitor water conditions in an area rich of marine species, as illustrated in Figure 1. Delivering information about critical species passages is essential for tracking migrations and preserving ecosystems. When data must be relayed beyond the underwater domain, connectivity can leverage terrestrial networks or other wireless systems, depending on coverage and requirements.

Underwater nodes must be smart and capable of promptly identifying marine species. By performing on-node classification, nodes dynamically adjust transmission parameters (e.g., modulation, transmission power, packet size, etc.) to minimize environmental disturbance. For example, rare species trigger low-rate modulation schemes for reliability purposes (e.g., BPSK) and smaller packet sizes to reduce noise pollution, while common species recognition allows for the use of higher spectral efficiency modulations (e.g., QPSK) for faster and larger data delivery. Transmission power is also tuned accordingly: lower power increases BER but avoids excess noise, while higher power increases throughput at the risk of harming marine life. Background noise from acoustic communications can cause behavioral changes, hearing damage, or physical harm to marine animals, as evidenced by documented cases linking mid-frequency sonar to cetacean strandings [14].

In our envisioned underwater monitoring system, the use of a distributed AI-based approach is essential due to the unique constraints of underwater environments. Traditional centralized systems, which rely on surface-based processing, cannot support real-time inference or dynamically adapt to the transmission parameters without overwhelming the limited bandwidth and exhausting energy resources. To address this, our design embeds lightweight AI models directly into sensor nodes, enabling on-node classification of marine species and real-time adjustment of transmission parameters (e.g., switching to BPSK modulation in case of monitoring of rare species). Each underwater node captures images, performs local species identification, and transmits only the classification result to the central station or to the neighbor node. This approach reduces data traffic, minimizes acoustic noise pollution, and aligns with ecological preservation goals. By decentralizing intelligence, the system avoids the pitfalls of centralized architectures, thereby avoiding bandwidth demands and risking harm to marine life through excessive acoustic traffic. Thus, distributed AI ensures scalability, energy efficiency, and environmentally responsible operation—critical requirements for sustainable underwater monitoring.

## 4. Channel Model

In this work, we adopt a widely used statistical model for underwater acoustic (UWA) channel characterization [15] (All the parameters used for UWA channel characterization are reported in detail at the following link: https://millitsa.coe.neu.edu/projects.html (accessed on 8 June 2025)) that captures both physical acoustic propagation effects and random variations, ensuring that our experiments reproduce realistic conditions. Two main types of channel variations are addressed:

- *Large-Scale Variations* due to the displacement of system components over many wavelengths, leading to slow, large-scale changes;
- *Small-Scale Variations* over short distances (a few wavelengths) and leading to fast fluctuations in the channel response.

In order to model large-scale variations coming from the displacement of the transmitter, the receiver, or due to the presence of reflective surfaces which cause changes in path delay and gain, the transfer function H(f) can be written as

(1) $$H\left(f\right)={\bar{H}}_{0}\left(f\right)\sum_{p}h_{p}e^{-j2\pi f\tau_{p}}$$

where $\tau_{p}$ and $h_{p}$ are the path delay and gain, respectively, where if the path length variation (location uncertainty) can be modeled as Gaussian, the path gain will be log-normally distributed. Also, the term ${\bar{H}}_{0}\left(f\right)$ accounts for the effects of path filtering, which is considered the same for all the paths.

Concerning small-scale variations, these are caused by scattering due to surface roughness, obstacles along the propagation path, and Doppler effects. The above phenomena cause rapid, short-term fluctuations in the received signal. Each propagation path is decomposed into “micropaths” with random delays and gains. In particular, the transfer function can be rewritten as

(2) $$H\left(f\right)={\bar{H}}_{0}\left(f\right)\sum_{p}\sum_{i}h_{p,i}e^{-j2\pi f\tau_{p,i}}$$

where $h_{p,i}$ is the intra-path gain and $\tau_{p,i}$ is the corresponding delay. Accordingly, a small-scale fading coefficient $\epsilon_{p}\left(f,t\right)$ can be considered, where

(3) $$\epsilon_{p}\left(f,t\right)=\frac{1}{h_{p}}\sum_{i\leq 0}h_{p,i}e^{-j2\pi f\delta \tau_{p,i}}$$

and thus,

(4) $$H\left(f\right)={\bar{H}}_{0}\left(f\right)\sum_{p}h_{p}\epsilon_{p}\left(f,t\right)e^{-j2\pi f\tau_{p}}$$

where $\tau_{p,i}=\tau_{p}+\delta \tau_{p,i}$.

Since the scattering points are separated by distances in the order of the typical λ values in relation to the low frequency traditionally used in acoustic underwater communication systems, the intra-path gains will be similar, but the phases will not. Thus, this will cause significant variations in $\epsilon_{p}\left(f,t\right)$.

The small-scale variations can be modeled as a complex Gaussian random process when a sufficiently high number of micropaths are present and the path amplitudes will exhibit a Ricean distribution.

Motion of the transmitter, receiver, and reflective surface results in Doppler shifts and spreads, which impact small-scale phenomena and can be modeled via the function ${\tilde{\epsilon }}_{p}\left(f,t\right)$:

(5) $${\tilde{\epsilon }}_{p}\left(f,t\right)=\epsilon_{p}\left(f,t\right)e^{j2\pi a_{p}\left(t\right)ft}$$

with $a_{p}\left(t\right)=\frac{v_{p}\left(t\right)}{c}$, taking into account the three types of elements that influence the Doppler, i.e., unintentional transmitter/receiver motion (drifting), intentional transmitter/receiver motion (vehicular motion), and effect of waves at the surface, with $v_{p}\left(t\right)$ being the velocity term and $\tau_{p}\left(t\right)=\tau_{p}-a_{p}\cdot t$.

## 5. Performance Analysis

In this section, we will detail the activity carried out to test the performance of the system. To this aim, first, we present the testing framework we have implemented; then, we discuss the numerical results.

### 5.1. Testing Framework

The overall framework considered in this paper has been implemented in GNU Radio 3.9 [16], while also exploiting external Mathworks Matlab [17] modules for modeling the underwater channel according to the Stojanovic underwater channel model [15] and as illustrated in the previous section. This module indeed accounts for multipath, frequency-dependent loss and Doppler shift, which typically characterize underwater channels. The simulation parameters used in the channel model are summarized in Table 1.

**Figure 2:**
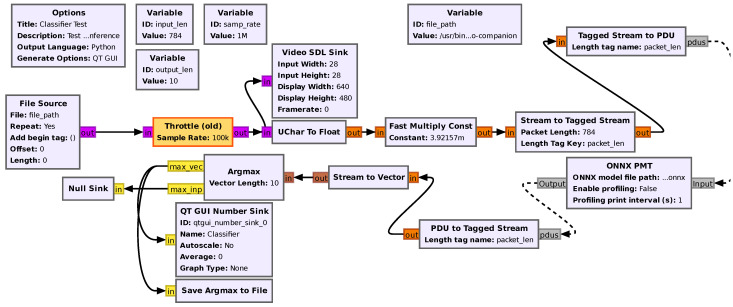
GNU Radio flowgraph for CNN inference.

The GNU Radio implementation of our system includes a classification module, as shown in Figure 2, which leverages convolutional neural networks (CNNs) for classification. CNNs, a type of deep learning model, are designed to process grid-like data such as images, videos, and time-series. They excel at identifying patterns and capturing spatial hierarchies using convolutional layers with filters.

Widely applied in tasks like image recognition, natural language processing, and video analysis, CNNs are trained through supervised learning on labeled datasets. Their key advantages include position-invariant feature detection, fewer trainable parameters compared to fully connected networks, and the ability to learn complex data representations. In Figure 2, the pre-processing of each image and conversion to an inference-acceptable format are highlighted. Then, this is fed to an ONNX-Runtime CNN block, and the post-processing of the result as a class label is executed to be later reused for the adaptation logic that triggers the transmission operations.

For simplicity, the standard MNIST dataset [18] has been used instead of a dedicated dataset of fish images. This, however, does not impact the applicability of the approach. In case of a different input, e.g., an RGB dataset, the larger images would increase inference latency, yet the transmission time and energy would remain unchanged because the system still sends only the compact class label. MNIST serves as a publicly available, well-controlled benchmark for validating the complete *sense–infer–transmit* pipeline. The same CNN architecture can be re-trained on RGB marine images using transfer learning [19], without requiring any modifications to the communication chain (Although classification energy increases with image size, a lightweight CNN model running on a Raspberry Pi 4, which typically operates at an active power between 2 W and 4 W, consumes approximately 1 mJ per inference for a 28 × 28 grayscale frame. When a lightweight CNN model, such as MobileNet or a custom architecture, is optimized through quantization or model compression techniques like pruning, the energy cost typically remains below 10 mJ for a 224 × 224 RGB frame. In contrast, transmitting the same 224 × 224 × 24-bit image which is approximately 150 KB over an EvoLogics 18/34 underwater modem at around 14 kbps across a 1 km link would require keeping the power amplifier active for roughly 90 seconds, resulting in energy consumption in the order of several hundred Joules. This is several orders of magnitude higher than the cost of local inference).

We used a trained ONNX inference model from [20], which consists of two convolutional layers, each followed by bias addition, ReLU activation, and a max-pooling layer. After the convolution and pooling stages, the output is flattened (reshaped) and passed through a fully connected (dense) layer, which outputs a 10-dimensional vector of class scores. The trained CNN inference model receives and performs the classification on the input image data. The output of the CNN model, which contains the classification probabilities, is converted back into a tagged stream using the PDU to Tagged Stream block. The result of the classification is the element with the highest predicted probability which is identified by an index.

In the rest of this section, we associate the numbers from 1 to 9 with 9 different classes of fishes, from the most common type (1) to the rarest and most vulnerable type (9). The second component of our framework, by exploiting the result of the classification process, selects the communication parameters such as packet length, gain, and modulation scheme to be utilized in the communication process. An example of the choice of the communication parameters for the nine classes is reported in Table 2 (In order to increase the reliability of the designed system and to cope with possible classification anomalies coming from the detection of new unclassified species, multiple back-up solutions can be designed. More specifically, in case of anomalies in data classification, additional data, for example, about vocalisms of mammals, can be employed so as to assess or correct classification results by means of integrated audio data. Another approach, which can also be used as a complementary mechanism to the above described one, is to transmit, in case of misclassification, the entire raw data while setting the transmission parameters in such a way as to preserve data delivery in any of the cases. The multiplicity of possible situations, which can be incurred in an unknown scenario like the marine one, clearly identifies the importance of figuring out a tradeoff between the preservation of reliability in data monitoring and the energy efficiency in data transmission).

Table 1 groups the classes assumed to be associated to species, based on the relevance and rarity of mammals. More specifically,

- *Classes 1–3* (associated to common species). In this case, modulations QPSK or BPSK are considered (with higher occurrence of QPSK modulation exhibiting higher spectral efficiency) with large packet length and high gain to maximize throughput when acoustic disturbance is not a concern.
- *Classes 4–6* (associated to vulnerable species). In this case, modulations QPSK or BPSK are considered (with higher occurrence of BPSK modulation exhibiting lower spectral efficiency) with medium packet length and medium gain to both preserve throughput and also cope with acoustic disturbance reduction due to the vulnerability of the considered species.
- *Classes 7–9* (associated to rare species). In this case, a fixed BPSK modulation is considered (exhibiting lower spectral efficiency but better reliability) with low packet length and low gain to cope with acoustic disturbance minimization because of the fragility of the considered species.

**Figure 3:**
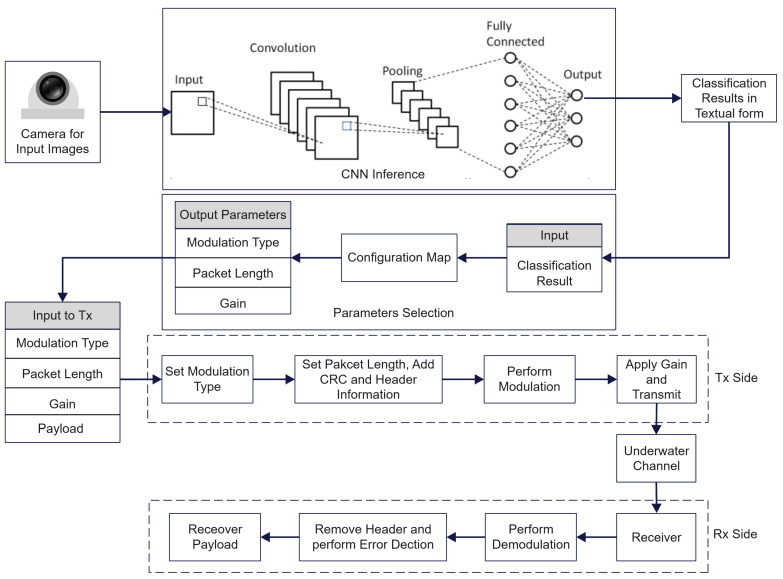
Components of the system.

Observe that our approach is general enough to account for other possible alternative ways of performing integrated sensing based on the semantics (i.e., on the meaning) of the data collected. As an example, if the underwater nodes are equipped with hydrophones, based on the sounds registered, if certain frequency components are present (e.g., those related to the vocalisms of certain species), appropriate sensors are activated, e.g., a camera sensor, and a similar classification can take place with the corresponding assignment of communication parameters. This is because such frequencies could be a hint for the presence of a group of rare mammals which, thus, can be visually tracked. A more general layout of the whole framework is shown in Figure 3, summarizing the processing chain: the sensed data (an image in our prototype) enter the CNN for inference; the resulting label is passed to a configuration map that chooses the modulation type, packet length and transmit gain; these parameters drive the transmitter blocks, so that data traverse the underwater channel and can be recovered by the receiver.

A software-defined radio (SDR) is a radio communication system where most key components that have traditionally been implemented in hardware are instead realized through software on a general-purpose computing platform. By using software to handle functions like modulation, demodulation, filtering, and signal processing, SDRs enable dynamic adaptation to new protocols, frequencies, and standards without requiring hardware changes.

One of the most widely used tools for SDR development is *GNU Radio* [16]. GNU Radio is a free and open-source development toolkit that provides signal-processing blocks to implement SDRs. It allows users to create radio systems through a graphical user interface (GUI) or by writing Python (3.12 version) scripts. The flexibility of GNU Radio lies in its modular architecture, where users can drag, drop, and connect pre-built signal-processing blocks to design complex radio systems.

GNU Radio is supported by a variety of hardware front-ends, such as universal software radio peripheral (USRP) devices and RTL-SDR dongles.

**Figure 4:**
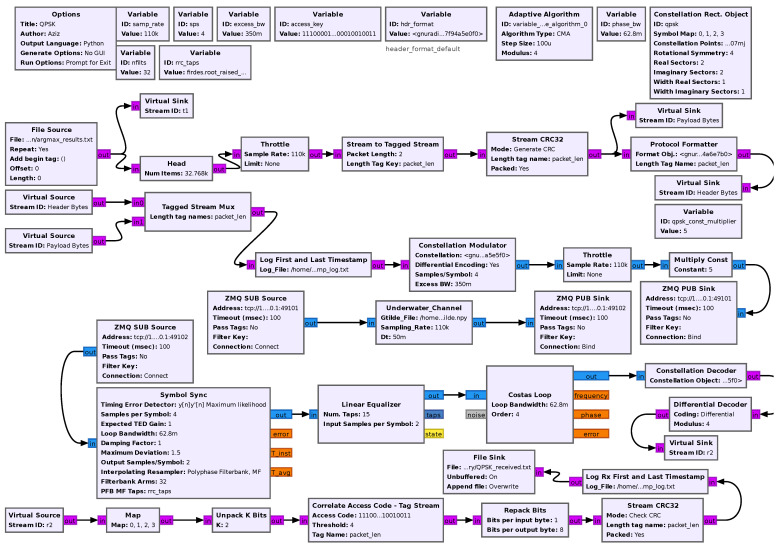
GNU Radio flowgraph for the communication system.

Concerning the GNU Radio implementation used for the SDR, sketched in Figure 4, within the communication architecture, the payload comprising the classification results is read and passed through the Stream to Tagged Stream block.

This converts the input data stream into defined packet sizes by creating boundaries, thereby enforcing the selected packet length. The CRC32 block generates a cyclic redundancy check (CRC) for the payload to ensure data integrity. A header section is generated for the payload. The Tagged Stream Mux combines the header and payload blocks and forwards them to the constellation modulator, followed by processing through a low-pass filter, fractional resampler, and gain controller. The modulation type and gain parameters are determined based on the previous choices, according to the classification results.

The modulated complex samples are then transmitted across the Stojanovic underwater channel model [15] and received at the receiver block. After passing through the resampler, automatic gain control (AGC), and filter blocks, clock synchronization is performed by the Symbol Sync block, and, after equalization (In our system, we prefer a simplified design; thus, we choose to consider a linear equalizer. However, different choices are possible, e.g., a decision feedback equalizer (DFE), which in any case, does not imply any change into the AI-driven controller behavior), phase correction is achieved using the Costas loop. Demodulation is carried out by the constellation decoder, which passes the decoded bytes to the differential decoder. The Correlate Access Code block searches for the specific 64-bit access key pattern, and the Repack Bits block reassembles the bits in most significant bit (MSB) style before forwarding them to the CRC Check block. The CRC Check block checks the accuracy of the received bits. Upon successful detection of the access key and verification of the CRC, the bits are repacked and the information is saved.

### 5.2. Numerical Results

**Figure 5:**
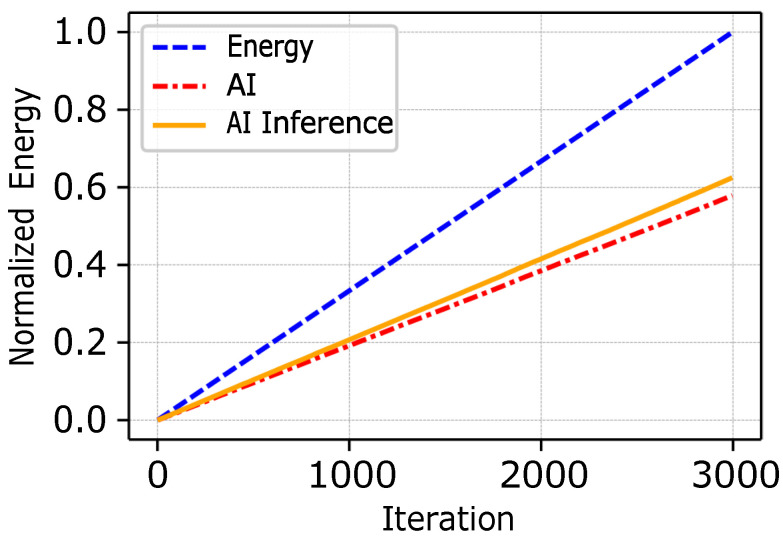
Energy consumption comparison.

Underwater acoustic networks have strict energy constraints, requiring adaptive strategies to optimize resource utilization without reducing functionality. As illustrated in Figure 5, we preliminarily studied the feasibility of the proposed strategy in terms of achievable energy efficiency when compared with methodologies not employing AI-driven design. Three approaches were considered, i.e., AI optimized when no effort in inference is accounted, AI with the consideration of on-node inference load, and a baseline approach. The baseline approach uses static communication settings such as default modulation, packet size, and transmission power, which result in consistently high energy consumption. The AI-optimized approach dynamically adjusts communication parameters based on classification results from inference systems, excluding the energy costs of inference itself, to isolate the impact of parameter adaptation. In contrast, the AI with the consideration of the on-node inference load approach integrates on-node lightweight models to locally classify species, accounting for the incurred computational energy costs while simultaneously performing real-time communication parameter optimization. We took the inference time details from [21] to account for the computational overhead introduced by classification. Although on-node inference introduces overhead, the combined benefits of reduced data transmission and context-aware optimizations yield net energy savings (in the order of about 40% upon increasing the number of iterations), as demonstrated in Figure 5. In this figure, each iteration on the *x*-axis corresponds to a complete communication cycle consisting of sensing, onboard classification using the CNN, and packet transmission over the acoustic link, i.e., 3000 repeated sense–infer–transmit operations. This underscores the viability of embedding inference-driven adaptability directly into nodes, balancing computational and communication costs for sustainable operation in dynamic underwater ecosystems. In the case of RGB images, the increased computational demand can be addressed using energy-efficient hardware accelerators, such as low-power GPUs or TPUs, in combination with lightweight architectures like MobileNet or EfficientNet. Quantization and pruning further reduce model complexity, while input dimensions can be controlled through simple pre-processing techniques such as cropping or resizing to regions of interest. Achieving high accuracy in marine environments largely depends on the quality and diversity of the training data. In our case, the ability of the model to generalize effectively to complex RGB images is significantly influenced by exposure to appropriate real-world underwater datasets. In contrast, when evaluated on the MNIST dataset, our lightweight 4-layer CNN model achieves an overall test accuracy of 99.1%, with individual class accuracies ranging from 98.6% to 99.7%.

To further evaluate the system performance, after assessing its suitability in terms of energy, we also considered other key performance metrics including BER, throughput, and delay.

#### 5.2.1. BER Analysis

**Figure 6:**
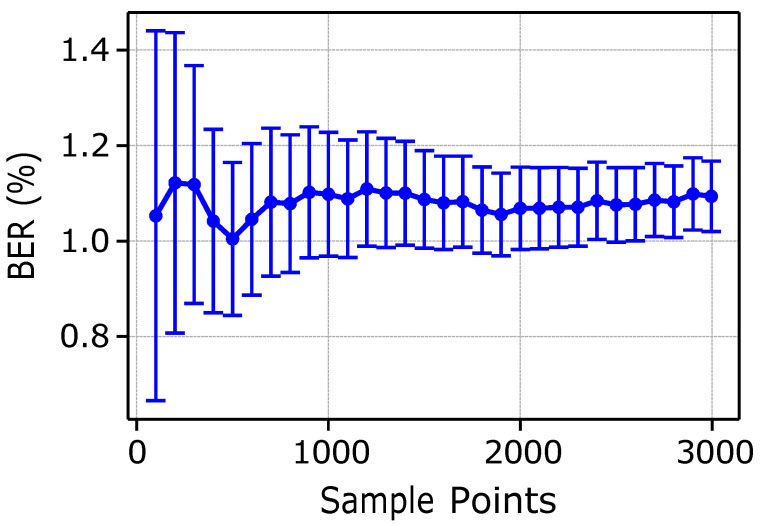
Mean BER over iterations.

In Figure 6, we show the mean BER (in %) as a function of the number of sample points, i.e., iterations, where each iteration corresponds to one complete communication cycle. Simulation results have been collected considering a T-Student distribution, with results providing an accuracy of 95%. Confidence intervals are shown through vertical bars in the plot.

**Figure 7:**
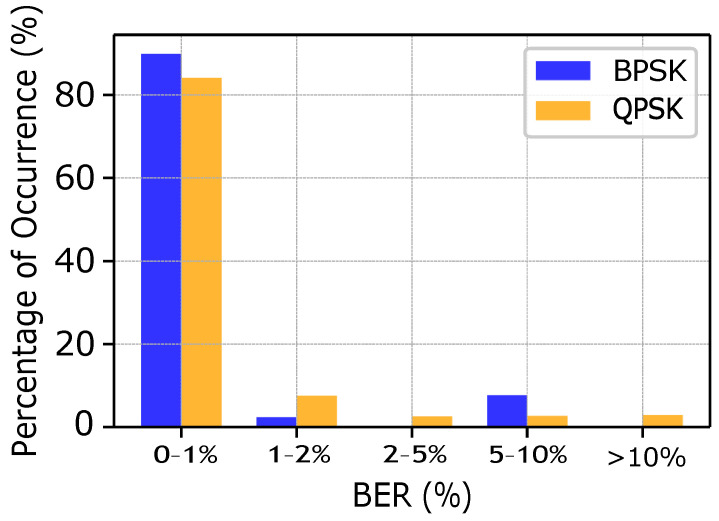
BER comparison for BPSK and QPSK.

A reduction in the confidence interval has required a minimum number of iterations in the order of at least 1500 samples. Note, however, that the BER remains around an average value of approximately 1.1%. In Figure 7, we report the detailed distribution of BER values for both the BPSK and QPSK modulation alternatives. The *x*-axis highlights the BER bins and the *y*-axis shows the percentage of occurrences within each bin. The majority of observed losses lie within the 0–1% range, which witnesses the reliability of the system, especially when considering the possibility to also exploit Forward Error Correction to speed up performance. Typically, in the case of BPSK, we observe a lower bit error rate as compared to the QPSK due to its intrinsic robustness in noisy conditions. In particular, in case of rare fish species, lower gain values and BPSK modulation are selected.

**Figure 8:**
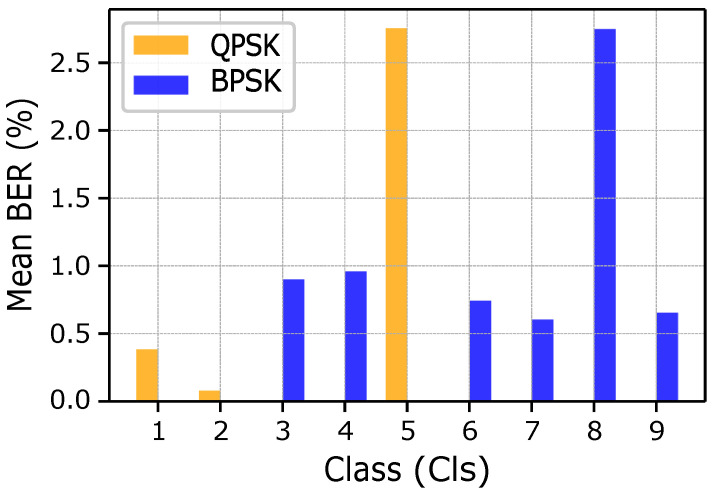
BER comparison for BPSK and QPSK for each class.

Similarly, in Figure 8, we show the histograms of the BER for different classes of fishes (1 to 9) and for both BPSK and QPSK modulations. In case of common fish species (low class values), no relevant environmental constraints emerge and, thus, higher gain and packet length are set. The difference here is due to the modulation type where classes 1 and 2 exhibit the minimum losses, as compared to class 3, which uses the BPSK and overpowered gain value. Similarly, for intermediate fish species from classes 4 to 6, the BPSK exhibits a lower BER as compared to QPSK, which requires more power and is less robust in noisy conditions. For the case of rare/vulnerable fish species, from classes 7 to 9, only BPSK is employed. Although the BER remains below 1% on average, occasional spikes occur due to the combination of low gain values and large packet sizes. These results show how modulation selection, gain optimization, and packet sizing choices are inter-related and can significantly influence system reliability under varying environmental and operational constraints.

#### 5.2.2. Throughput Analysis

**Figure 9:**
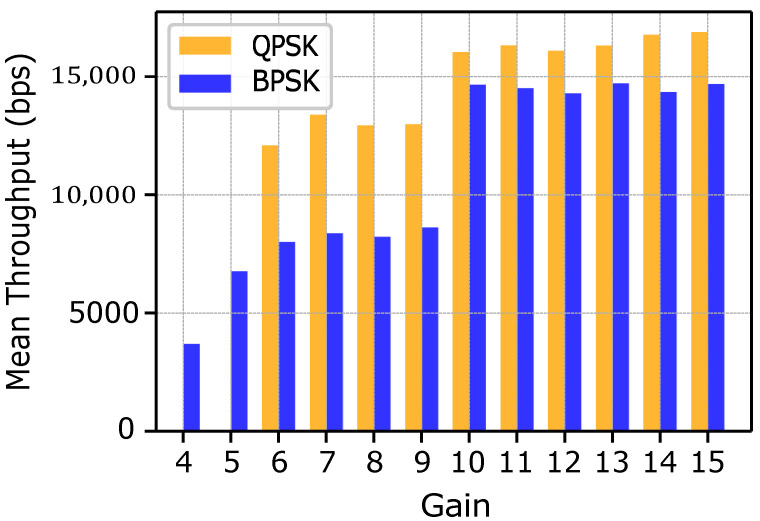
Throughput comparison for BPSK and QPSK at different gain values.

Figure 9 illustrates the throughput (in bps) achieved both in cases of BPSK and QPSK modulations across various gain values, corresponding to different categories of fish species.

For rare species, low gain values combined with small packet lengths result in comparatively modest throughput levels, ranging from approximately 3700 bps to 5000 bps. Although these conditions represent a challenging scenario, the observed performance underscores BPSK robustness and ability to work even under limited power amplification. In the case of intermediate fish species, both BPSK and QPSK operate within gain ranges of approximately 5 to 9 and 6 to 9, respectively. Due to the increased packet size and slightly improved gain, BPSK throughput rises sharply in the initial portion of this range and then stabilizes. By contrast, QPSK reaches a throughput of nearly 12,000 bps at the lower end of its gain range and stabilizes around 13,000 bps as gain increases further. For common fish species, the highest gain values are employed to achieve substantially improved throughput.

BPSK stabilizes at approximately 14,250 bps, although occasional declines occur due to increased bit losses at very high gains. Meanwhile, QPSK initiates this segment around 16,000 bps and continues to show an upward trend, demonstrating its ability to leverage higher gain values and larger packet sizes for further throughput enhancement.

#### 5.2.3. Delay Analysis

**Figure 10:**
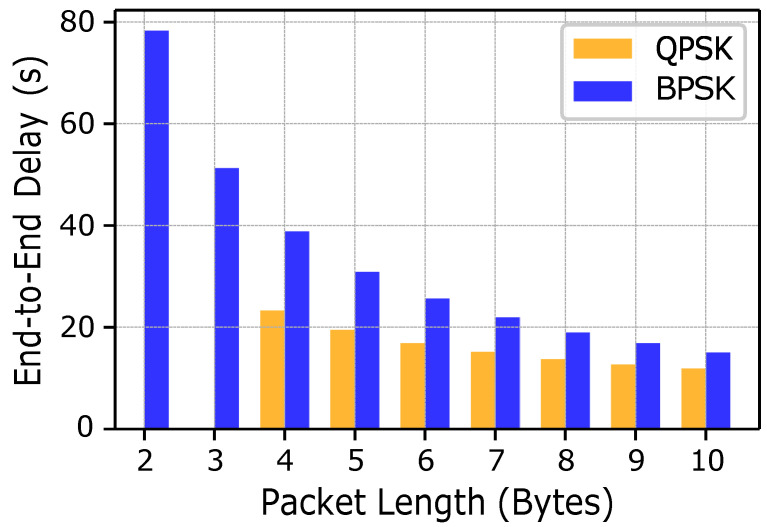
End-to-end delay comparison for BPSK and QPSK.

Figure 10 shows the end-to-end delay (in s) as a function of packet length (in bytes). Both BPSK and QPSK follow the general trend of decreasing delay as the packet length increases. However, BPSK consistently requires approximately twice as much time as QPSK. Smaller packets incur proportionally higher overheads than larger packets, so increasing packet size allows more useful information to be carried per packet and, thus, improves efficiency and reduces delay. Furthermore, the higher spectral efficiency of QPSK enables it to transmit more bits per symbol than BPSK, which further shortens the end-to-end delay. For packet lengths of 2 and 3 bytes (representing rare fish species), only BPSK modulation can be chosen; however, it provides the advantage of greater robustness at lower gains and in noisy conditions. For the remaining fish classes, the delay trend aligns closely with the general behavior described above.

## 6. Conclusions

In this paper, we introduced a solution for underwater communications that adaptively modifies communication parameters to minimize disturbance to the natural environment while enhancing energy efficiency. A key characteristic of the proposed approach is that all core operations, including environmental sensing, classification, and communication adaptation, are executed in real-time onboard the software-defined radio platform using the GNU Radio framework. By enabling localized inference and adaptive transmission, the system reduces data volume and optimizes energy consumption. The approach was implemented and validated in a simulated environment, demonstrating its potential for sustainable and energy-aware underwater communications.

As part of our future work, we plan to conduct real-world field trials to evaluate the framework under actual underwater acoustic conditions, including dynamic channel behavior, mobility, and environmental noise. These experiments will provide critical insights into the system’s robustness and scalability and will help refine the adaptive transmission strategies for practical deployment.

## Figures and Tables

**Table 1:** Simulation parameters for underwater acoustic channel modeling.

Sr. No.	Parameter (Symbol)	Value
1	Operating Frequency ($f_{0}$)	10–20 kHz
2	Bandwidth (*B*)	10 kHz
3	Depth ($h_{0}$)	100 m
4	Transmitter Depth ($h_{t}$)	20 m
5	Receiver Depth ($h_{r}$)	50 m
6	Channel Distance (*d*)	1000 m
7	Spreading Factor (*k*)	1.7
8	Speed of Sound in Water (*c*)	1500 m/s
9	Speed of Sound in Bottom ($c_{2}$)	1200 m/s
10	Variance of Surface Fluctuations ($\sigma_{s}^{2}$)	1.125
11	Variance of Bottom Fluctuations ($\sigma_{b}^{2}$)	0.5625
12	Surface Variation Amplitude ($A_{w}$)	0.05
13	Surface Variation Frequency ($f_{w}$)	0.01
14	Number of Intra-Paths per Path ($S_{p}$)	20
15	Mean Intra-Path Amplitude ($\mu_{p}$)	0.025
16	Variance of Intra-Path Amplitude ($\nu_{p}$)	$10^{-6}$

**Table 2:** Configuration map table.

Class Labels	Gain	Packet Length	Modulation
1–3	[10–15]	[7–10]	QPSK
	[10–15]	[4–6]	QPSK
	[10–15]	[7–10]	BPSK
4–6	5	[4–6]	BPSK
	[6–9]	[4–6]	QPSK
	[6–9]	[4–6]	BPSK
7–9	4	2	BPSK
	4	[2–3]	BPSK
	5	3	BPSK

## Data Availability

Data are contained within the article.

## Abbreviations

The following abbreviations are used in this manuscript:

AI	Artificial Intelligence
SDR	Software-Defined Radio
BER	Bit Error Rate
UASN	Underwater Acoustic Sensor Network
BPSK	Binary Phase Shift Keying
QPSK	Quadrature Phase Shift Keying
IoUT	Internet of Underwater Things
MCU	Microcontroller Unit
CNN	Convolutional Neural Network
GUI	Graphical User Interface
USRP	Universal Software Radio Peripheral
UWA	Underwater Acoustic
CRC	Cyclic Redundancy Check
AGC	Automatic Gain Control
MSB	Most Significant Bit
DFE	Decision Feedback Equalizer
